# Pork Consumption Frequencies, Attitudes and Sensory Acceptance of Traditional Products in Lithuania

**DOI:** 10.3390/foods11203292

**Published:** 2022-10-20

**Authors:** Violeta Razmaitė, Rūta Šveistienė, Virginija Jatkauskienė, Artūras Šiukščius

**Affiliations:** Animal Science Institute, Lithuanian University of Health Sciences, R. Žebenkos 12, LT-82317 Baisogala, Lithuania

**Keywords:** pork sausages, backfat, local breeds, blind liking

## Abstract

Finding a niche for the wider use of local pigs highlighted the need for information about consumer attitudes regarding pork and traditional products and the acceptability of fatter meat. With the aim to ascertain pork consumption frequency and Lithuanian consumer attitudes towards traditional pork products, as well as acceptability of traditional sausages from the meat of Lithuanian White pigs, a questionnaire-based survey and consumer sensory tests were conducted. A total of 136 meat consumers participated in the study. Respondents reported that they consume fresh or processed pork from 1 to 10 times weekly. Male respondents were more familiar with Lithuanian local pig breeds, while female respondents demonstrated knowledge of pork products. Boomer generation (1946–1964) respondents mostly (χ^2^ = 29.53, df = 10, *p* < 0.001) had pork at home compared with the respondents of younger generations. There were no significant differences in the blind sensory acceptance between sausages made in a traditional way and cold-smoked with different quantity of salt and commercial sausages of premium quality, while conventional hot-smoked sausages had lower (*p* < 0.001) overall acceptance. The highest (*p* < 0.005 and *p* < 0.01, respectively) acceptance for salt reduction in traditional sausages was demonstrated by the X generation (1965–1980) consumers compared with older boomer and subsequent Y (1965–1980) generations.

## 1. Introduction

Meat consumption has a crucial role in human evolution and nowadays is an important component of balanced diet [1,2] and part of food cultures [3]. Pork remains as the most commonly consumed meat, and among meat products, pork and its products occupy an important place [4,5]. Pigs were the animals that were easier to hide during the attacks of enemies in ancient times. Additionally, due to their rapid reproduction and growth, pigs have been considered a symbol of prosperity, and Europeans have created many different breeds [6].

Since the middle of the twentieth century, consumption of meat and other products of animal origin has drastically increased, whereas plant-based foods (vegetables, fruit, pulses, nuts, grains, seeds) have reduced in percentage [7]. Despite meat nutritional richness, the overconsumption of meat has been considered a disease; promoting food and meat has increasingly become a subject of controversies relating to health and safety [8,9]. However, research has started to demystify the negative health issues, and although on the one side, there is evidence accumulating that meat itself is not a risk factor, and rather the risk stems from the consumption of processed meats, but on the other side, health effects associated with processed meat consumption are still equivocal and depend on consumption level [10]. Mankind started to produce meat products with the aim to extend self-life and enhance flavour, and meat has been transformed through salting, curing, cold and hot smoking to different products and now there is a wide range of products that differ from each other in terms of meat type, salt and fat content, the processing method applied and eating occasion [4,11,12,13]. Companies in the meat sector develop and launch new products all the time. However, among all different products there are traditional food products—an important element of culture, identity, and heritage of society. Traditional products were defined as the result of traditional production practices, gastronomic heritage, and culinary habits representing the identity and culture of specific geographical areas and territorial dimensions. Traditional products also mean their usage for a long time period showing transmission between generations [14,15]. These products can contribute to the development and sustainability of rural areas, protecting them from depopulation, entailing substantial product differentiation potential for producers and processors and providing large variety in food choice for consumers [14,16]. Therefore, production and consumption of traditional pork products can be a way to preserve local pig breeds [17,18,19]. However, production of traditional food still relies on traditional manufacturing practices, often with low competitiveness and poor efficiency [20] or is left entirely as home-made production. Therefore, traditional food producers have been recommended to extend their skills in modern production techniques, management and marketing, as well as in promoting the aspects of their products related to nutritional and health issues [20]. In order to maintain and even increase their market share, traditional food products (TFP) need to be improved by introducing innovations that fulfil the consumers’ demand for better TFP, including health, safety and taste. Although many of consumers have a positive attitude towards local and traditional food products, but they also have different doubts about traditional food products and express insecurity about health issues [21]. Many authors have reported that the highest levels of acceptance are found for innovations that reinforce the traditional character of the product, guarantees the origin of the raw material [22,23], and provide benefits from improving negative attributes such as reduction of fat or salt content [23,24].

Although Lithuanian researchers are gradually getting involved in consumer food preferences and choice studies [25], the data on consumer attitudes and acceptability of pork and traditional pork products are scarce. Lithuania has two local pig breeds: Lithuanian Indigenous Wattle and Lithuanian White pigs. However, both these breeds are at high risk of extinction. Primarily, Lithuanian White pigs, as a more productive breed displaced ancient Lithuanian Indigenous Wattle pigs and, afterwards, a drastic decline in the numbers of small farms and reckless pig crossbreeding resulted in a significant reduction of the purebred Lithuanian White pig population. Although nowadays purebred individuals of the above breeds are still retained and both breeds are under a conservation program, the call for a wider usage of these breeds is required. Therefore, finding a niche for wider use of local pigs highlighted the need of information about consumer attitudes regarding pork and traditional products and the acceptability of fatter meat.

The aim of the present study was to determine pork consumption frequency, Lithuanian consumer attitudes towards traditional pork products, and acceptability of traditional sausages from Lithuanian White pig meat with different salt content.

## 2. Materials and Methods

### 2.1. Participants and Data Collection

To evaluate pork consumption frequency and attitudes, in addition to preferences regarding traditional Lithuanian pork products, a paper questionnaire-based survey and consumer sensory tests were conducted. A total of 136 individuals, including 91 women (25–71 years) and 45 men (20–72 years) participated in the study. All the respondents were meat consumers. Each respondent completed the questionnaire. The questionnaire was subdivided into three main sections. The first section included questions related to the frequency of pork consumption and consumer knowledge about local breeds and products produced from the meat of local pig breeds. The second section included questions about traditional and new products. In this section, a 9-point Likert scale was formed by the following categories: “disagree very strongly”, “disagree strongly”, “disagree moderately”, “disagree slightly”, “neutral: neither agree nor disagree”, “agree slightly”, “agree moderately”, “agree strongly” and “agree very strongly.”

The end of the questionnaire assessed the following sociodemographic variables: gender, year of birth, income, education, number of persons living in the household.

After the respondents had completed the questionnaire, they were asked to evaluate the products. Blind liking (perceived sensory acceptability) was used for the evaluation of products. During the sensory test consumers received samples of 4 sausages and were asked to taste them in a pre-defined random order. The samples of products were coded, no product information has been provided for the participants. Between two tastings, consumers were asked to rinse their mouths with water and eat a piece of bread. The participants tested and evaluated four products using a 9-point scale (from 1 = dislike extremely to 9 = like extremely) according to their perceived taste and overall acceptability as described by the authors who evaluated traditional products in other countries [26].

### 2.2. Products

Two types of cold-smoked traditional (T) and traditional innovative (IT) sausages were manufactured following the traditional recipe in the facilities of the Department of Food Technologies, Kaunas University of Applied Sciences. The ingredients consisted of 75% minced lean pork and 25% of subcutaneous backfat from the Lithuanian White pig breed, salt, black pepper and garlic. To improve their healthiness, the innovation proposed was aimed to reduce the salt content in IT sausages. The T consisted of 3%, whereas IT consisted of 2% salt. The made sausages were ripened for a day and then naturally cold- (+25 °C) smoked using alder wood for 3 days.

Additionally, two other types of sausages were purchased to compare these traditionally made sausages with commercial products that are available in the market, i.e., premium cold-smoked pork “Skilandinė” and hot-smoked sausages made from conventional pig breeds in an industrial plant.

### 2.3. Data Analysis

The data analyses were performed using IBM SPSS Statistics for Windows, Version 27.0. Armonk, NY: IBM Corp software. Descriptive analyses were performed on pork consumption frequencies and consumer attitudes and preferences regarding pork and pork products. The differences in the attitudes, preferences and consumption were compared using chi-square statistics. The preference data were subjected to the analysis of variance in repeated measures of the general linear (GLM) procedure. For the effect of consumer gender and generation/age evaluation, the GLM univariate model included fixed factors of consumer gender and generation. The differences were regarded as significant when *p* < 0.05, but the differences of 0.05 < *p* < 0.10 would be considered as trends after applying LSD tests.

## 3. Results and Discussion

### 3.1. Consumer Surveys

The characteristics of the respondents are presented in Table 1. The sample includes a higher proportion of females (66.9%) than males. There are more women in Lithuania and they are more often responsible for food in the families. As a total population, the respondents also varied in other sociodemographic characteristics, psychological factors, individual attitudes and criteria.

**Figure 1:**
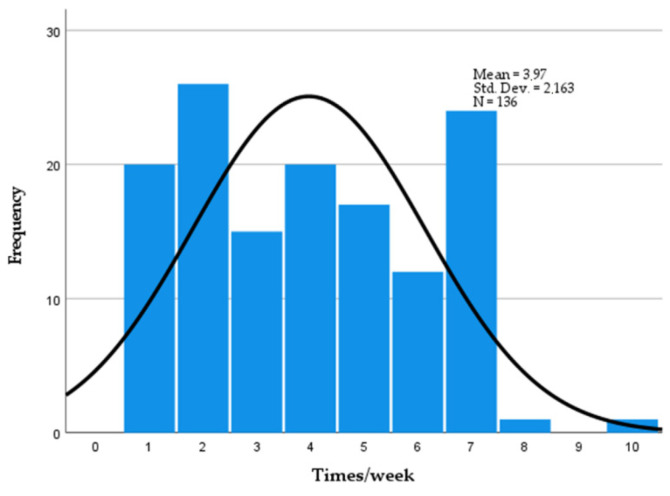
Pork consumption per week.

Diversity was observed in the respondent answers using the questionnaire survey. Globally, poultry consumption overtops that of pork [27,28]; however, in Lithuania from the total amount of 69 kg of meat per capita, as much as 40 kg is pork [29], and Lithuanians are mentioned even by foreign authors as hearty pork eaters [27]. Pork consumption within Lithuanians varies widely. The respondents reported that they consume fresh or processed pork from 1 to 10 times weekly (Figure 1). However, most frequently Lithuanians consume pork two, seven and four times weekly (19%, 17% and 14.7%, respectively).

**Figure 2:**
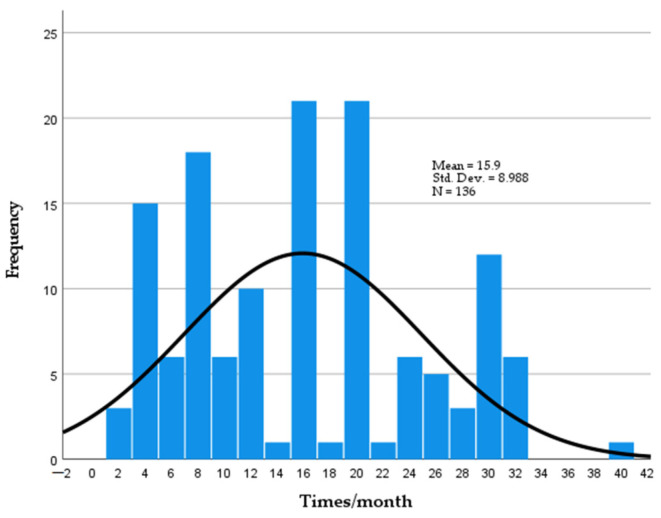
Pork consumption per month.

This study confirmed that consumer habits have not changed in comparison with our previous study [30], which showed that 17% of people consume pork daily. Most frequently, pork consumption was indicated four, eight, sixteen and twenty times monthly (9.6%, 12.5%, 10,3% and 15.4%, respectively). Only 2.2% and 3.7% respondents indicated pork consumption pork 2 and 40 times monthly, respectively (Figure 2).

In total, 69.9% of respondents answered that the last time they consumed pork was a day before. The review of published papers explicated seven consumer credence of food categories: health-related components, organic, origin, brands, production method-related, ethics-related and descriptive food names and ingredients [31]. Gender also appears to be an influential moderating factor when it comes to responses about healthy food, however, younger consumers are less concerned about health and more interested in taste than older consumers. The country-of-origin effect is evident as a clear tendency for domestic produce to be favoured over imports, and more local or regional food to be favoured over food with less specified origins [31]. Female consumers in European countries reported eating pork of all categories significantly less frequently than males [3,32,33,34]. In the present study, the differences between genders on pork consumption frequencies were not significant and this was in agreement with Rosenfeld and Tomiyama [35], however, other authors [36] have reported gender differences on attitude towards red meat, but these differences were quite small, explaining lower variance between genders than age or cultural factors. The Pearson chi-square test showed that the respondents from the older boomer (1946–1964) generation mostly reported eating pork a day before (χ^2^ = 35.14, df = 16, *p* < 0.001). Despite rare monthly pork consumption by some persons, all of them remembered having consumed pork during the last week. Additionally, 19.3%, 6.7%, 2.5% and 1.7% of respondents reported that they consumed pork or pork products two, three, four and six days ago, respectively. The answer to the question whether they have pork at home now resulted in 83.1% of respondents admitting to having pork at home, 13.2% of the respondents were indifferent and did not know and only 3.7% pointed out that they had no pork at home. Boomers were most frequent (χ^2^ = 29.53, df = 10, *p* < 0.001) pork holders at home compared with the respondents from younger generations. Most of the respondents (41.2%) indicated that pork is usually purchased pork at the traditional market and this is in contrast with the findings in other five European countries [32] where almost half of the fresh pork purchases were made in supermarkets, and another half at local butchers, although the distribution varied considerably between the countries. In Lithuania, supermarkets are another also popular place to buy pork. Since there are not many specialized butchers in Lithuania, 13.2% of the respondents buy pork from them and 17.6% of the respondents reported that they buy pork from abattoirs or directly from small farms.

Consumer preferences, attitudes and intensions not only towards domestic pork production but also towards the specific breeds that are used for pork and traditional products could be a tool for pig breed conservation policy as it is in other countries [17,18,19]. In addition, 58.1% of the respondents indicated that they had seen or heard about Lithuanian pig breeds. Male respondents knew about the local breeds better (χ^2^ = 10.71, df = 1, *p* < 0.001) than female respondents. However, females indicated higher variety (χ^2^ = 4.69, df = 1, *p* < 0.05) of known products compared with males. The age (generation) of the participants also affected the knowledge about the breeds. The respondents from boomer (1946–1964) and X (1965–1980) generations had better (χ^2^ = 13.03, df = 5, *p* < 0.05) knowledge about the breeds and indicated seeing them more frequently (χ^2^ = 12.42, df = 5, *p* < 0.05) than the younger Y (1981–1996) generation. The question about the cognition of local breeds was answered as follows: only 0.7% of the respondents indicated Lithuanian Indigenous Wattle pigs, 11.8%% of the respondents indicated both breeds and 8.5%-Lithuanian White pigs.

The published results on the consumer acceptance of Italian dry-cured hams revealed that consumer liking was more affected by the specific Protected Designation of Origin (PDO) technology than by the genetic type. Toscano ham was the most preferred and most familiar product among Tuscan consumers, indicating that familiarity with the product was the best driver of dry-cured ham preference [37]. Moreover, the results of the study conducted in Hungary also indicated that Protected Geographical Indication (PGI) can generate value to consumers exceeding that of the leading private brand [38], and there is reason to believe that Lithuanian consumers will also appreciate traditional products made from Lithuanian pig breeds.

In the present survey, sausages (24.4%), backfat (23%) and ham (10.6%) were most often mentioned as familiar pork products. Only 0.7% of the respondents mentioned “Skilandis”, a national heritage product of exceptional quality and the most expensive one, as well as meat jelly. Since backfat was mentioned almost as often as sausages, it suggests that certain numbers of even more fatty pigs may also have a niche for their use. Sausages, backfat and ham as well as “Skilandis” are traditionally cold-smoked products. Pork smoking in Lithuania has been used as a preservation method since ancient times and nowadays has remained popular. Commercially, an alternative method such as application of liquid smoke is used. Although there are publications [39,40] showing the positive properties of liquid smoke, consumers trust and appreciate traditional natural cold-smoking more. Much of the available research on smoked meats relates to the potential carcinogenic compounds (polycyclic aromatic hydrocarbons PAH) produced during preparation. Different studies reported that wood type has a significant effect on the final PAH content of the smoked meat [41,42]. The research carried out in Latvia indicated that pork smoked with apple tree and alder, the tree which is widely used in Lithuania, contained the lowest amounts of PAH [41].

In total, 45.6% of the respondents strongly and very strongly agreed that they are constantly consuming new products and only 10.3% of them do not trust new foods and do not want to try (12.5%) unknown food (Table 2). Boomers were most (χ^2^ = 55.75, df = 40, *p* < 0.05) reluctant to try unknown food. Additionally, 33.8% and 11.0% of respondents strongly and very strongly disagreed and agreed, respectively, that they were afraid of eating things they had never eaten before. However, at dinner parties women were more likely to try the new foods (χ^2^ = 15.25, df = 8, *p* = 0.054) compared with men. Although a very similar number of the respondents disagreed (22.7%) and agreed (26.5%) that they eat almost everything, 80.8% indicated the importance of the food they eat. From moderate to very strong belief, the respondents agreed that traditional products are strongly associated with the past (55.2%), specific localities or countries (61.8%), however, the associations of the products with the memories from childhood were much weaker (47.8%). The assertation that traditional products are frequently consumed and associated with specific celebrations or seasons was strongly and very strongly supported by 36% and 30.9% of the respondents, respectively.

A significantly larger proportion of the respondents strongly associated traditional products with gastronomic heritage (60.3%) and were of opinion that these products are produced following recipes passed from generation to generation (43.4%). This opinion was exceptionally supported (χ^2^ = 73.18, df = 35, *p* < 0.001) by older boomer and X generations. Only a half (50.7%) of current Lithuanian consumers associate traditional products strongly and very strongly with their production in a domestic setting or by artisans. The consumers have also similarly agreed that traditional products help the local economy (50.7%) and are environmentally friendly (48.4%). Moreover, 69.9% and 65.4% of the respondents, including their answers from moderate to very strong, agreed that traditional products possess distinctive and positive sensory merits and are safe, respectively. The claim that traditional products are low quality products was strongly and very strongly denied by 50.8% of consumers, however, 8.1% of the respondents agreed. In addition, 3.2% of Lithuanian consumers strongly and very strongly agreed that traditional products are fat and not healthy, however, 25% of other respondents strongly and very strongly disagreed with such an opinion and most of them (37%) slightly and moderately agreed with higher fatness of traditional Lithuanian products. The opinions of genders on this issue were different. Women were more likely to disagree, while men were more likely to agree (χ^2^ = 18.04, df = 8, *p* < 0.05) that traditional products are fat and unhealthy. Although the consumers mentioned only three products, i.e., sausages (28.1%), backfat (27.5%) and ham (17%) as traditional, they strongly disagreed (31.6%) that traditional pork products have narrow assortment of varieties and flavours. Additionally, 41.2% against 8.1% of the participants also strongly and very strongly disagreed and agreed, respectively, that traditional pork products have unattractive appearance.

The changes in sensory and consumer research have more emphasis on health and wellness [43], therefore most of recent innovations in processed meats focus on healthier reformulations through reducing negative constituents or adding health beneficial ingredients [24]. The findings of Vanhonacker et al. [23] illustrated that consumers are open towards innovations in traditional food products. The highest levels of acceptance were found for innovations that reinforce the traditional character of the product (a label that guarantees the origin of the raw material) or provide benefits from improving negative attributes associated with the traditional character of foods such as reduction of fat or salt content [14,22,23]. Research of other authors indicate that consumers showed a preference for reduced salt and fat over non reduction [24].

### 3.2. Sensory Evaluation

There were no significant differences in the blind sensory acceptance between the sausages made in a traditional way, cold-smoked with different quantity of salt and commercial sausages of premium quality, while conventional hot-smoked sausages had lower (*p* < 0.001) overall acceptance (Table 3).

The absence of significant differences in the evaluation of traditional sausages and commercial ones of premium quality are consistent with the blind evaluation of Cinta-Senese sausages in Italy [26]. The gender of the participants did not affect the acceptance of the tested products, except for a negligible tendency (*p* = 0.095) in the worse evaluation of hot-smoked conventional sausages by male participants. The biggest differences were found in the acceptance of innovative traditional sausages with a reduced salt amount between the consumers belonging to different generations. The highest (*p* < 0.005 and *p* < 0.01, respectively) acceptance for salt reduction in traditional sausages was demonstrated by the participants of X (1965–1980) generation compared with older boomer (1946–1964) and younger Y (1965–1980) generations. The obtained results suggest that Lithuanian consumers are in the habit of eating commercial conventional products as well as traditional products and the tastes of these products make the basis of commercial industrial recipes.

## 4. Conclusions

The study confirmed that pork and traditional pork products are popular and frequently used in Lithuania. The differences in pork consumption and in attitudes towards pork and pork products between consumers’ gender and age were not significant. Although male consumers are more aware of local pig breeds, female consumers demonstrate good knowledge of products and, moreover, at dinner parties women are more likely to try new foods (if present) compared with men. Women were also more likely to disagree, while men were more likely to agree that traditional products are fat and unhealthy.

The majority of the respondents of the oldest generation stated that they have pork at home and are reluctant to try unknown food, and also strongly associate traditional products with gastronomic heritage and have the opinion that these products are produced following the recipes passed from generation to generation. However, the age of the consumer did not affect the acceptability of the products, except for the innovations with reduced salt content. Even in this case, the reduction of salt was most favourably accepted by the middle age generation. The results of the study showed that consumer tastes are taken into account in industrial production and product recipes. The fact that backfat was mentioned almost as often as sausages, and the claim that traditional products are low quality products was strongly and very strongly denied by 50.8% of consumers, allows us to draw the conclusion that certain numbers of even fatter local pigs may also have a niche for their use. 

## Figures and Tables

**Table 1:** Sociodemographic characteristics of respondents.

Categories	Sociodemographic Factors	%
Gender	Female	66.9
	Male	33.1
Generation/Age	Boomers (1946–1964)	37.7
	X (1965–1980)	33.8
	Y (1981–1996)	28.5
Household with children <12 years		25.0
Number of children up to 12 years in a household (persons)	1.41	
Household size (persons)	2.73	
Household perception of the monthly net income compared to the average	Far below average	12.5
	Below average	24.3
	On average	33.8
	Above average	22.1
	Far above average	5.9
	Not known	1.5

**Table 2:** Frequency of respondents’ answers (%) related to affirmations about pork products.

Affirmation	Dis-Agree Very Strongly	Dis-Agree Strongly	Dis-Agree Moderately	Dis-Agree Slightly	Neutral	Agree Slightly	Agree Moderately	Agree Strongly	Agree Very Strongly
	1	2	3	4	5	6	7	8	9
Are anchored to the past	0.7	5.9	1.5	2.2	17.6	16.9	14.0	33.8	7.4
Are tied to specific localities, regions or countries	0.7	5.9	1.5	2.2	12.5	15.4	20.6	33.8	7.4
Evoke strong memories of childhood	2.2	6.6	1.5	2.2	18.4	21.3	8.8	29.4	9.6
Are a part of an area’s gastronomic heritage	0.7	1.5	0.7	0.7	7.4	14.0	14.7	44.1	16.2
Are frequently consumed products	1.5	8.1	2.9	7.4	14.7	14.0	15.4	27.9	8.1
Are associated to specific celebrations or seasons	1.5	11.8	2.2	5.1	18.4	18.4	11.8	24.3	6.6
Are produced following recipes passed from generation to generation	0.0	2.9	0.7	4.4	8.1	22.1	18.4	33.8	9.6
Are produced in a domestic setting or by artisans	0.0	2.2	1.5	5.9	6.6	22.8	10.3	39.7	11.0
Help local economy	2.9	2.9	0.0	2.2	10.3	19.1	11.8	41.9	8.8
Are environmentally friendly	2.9	3.7	0.0	5.1	25.0	14.7	13.2	30.1	5.1
Possess distinctive and positive sensory merits	0.7	0.7	0.0	0.7	11.0	16.9	19.1	41.2	9.6
Are safe	0.7	0.7	0.7	2.9	17.6	11.8	16.9	41.9	6.6
Are of low quality	0.7	14.0	36.8	9.6	8.1	14.0	6.6	2.2	8.1
Are not healthy and contains higher fat amount	4.4	20.6	5.1	8.8	14.0	19.9	14.0	10.3	2.9
Has narrow assortment of varieties and flavours	2.2	29.4	8.1	7.4	19.9	13.2	10.3	8.8	0.7

**Table 3:** Acceptability scores of blind product evaluation and effects of consumer gender and generation on acceptance of traditional and commercial products.

Product	Overall	Gender		SED	Generation/Age			SED	*p*-Value	
		Female	Male		B	X	Y		Gender	Age
T	6.72 ^e^	7.00	6.77	0.563	6.71	6.86	6.60	0.321	0.677	0.849
IT	6.76 ^e^	7.21	6.48	0.588	6.35 ^a^	7.22 ^b,c^	5.74 ^d^	0.336	0.217	0.044
CP	6.46 ^e^	6.54	7.15	0.622	6.30	6.26	7.30	1.361	0.334	0.404
Hot-smoked	5.63 ^f^	5.47	4.43	0.620	5.49	5.44	6.27	0.354	0.095	0.110

T-traditional cold-smoked; IT-innovative traditional cold-smoked; CP-premium quality commercial product, cold-smoked; *p* values of GLM LSD tests for groups were significantly different at ^a–b^ *p* ˂ 0.05; ^c–d^ *p* ˂ 0.01; ^e–f^ *p* ˂ 0.001.

## Data Availability

Data is contained within the article.
